# Fourfold daily growth rate in multicellular marine alga *Ulva meridionalis*

**DOI:** 10.1038/s41598-020-69536-4

**Published:** 2020-07-28

**Authors:** Masanori Hiraoka, Yutaro Kinoshita, Motoki Higa, Shuntaro Tsubaki, Alvin P. Monotilla, Ayumu Onda, Akinori Dan

**Affiliations:** 1 Usa Marine Biological Institute, Kochi University, 194 Inoshiri, Usa, Kochi, Tosa 781-1164 Japan; 2 Science Department, Graduate School of Integrated Arts and Science, Kochi University, 2-5-1 Akebono-cho, Kochi, 780-8520 Japan; 3 Laboratory of Plant Ecology, Faculty of Science and Technology, Kochi University, 2-5-1 Akebono-cho, Kochi, 780-8520 Japan; 4 Department of Chemical Science and Engineering, School of Materials and Chemical Technology, Tokyo Institute of Technology, 2-12-1 Ookayama, Meguro, Tokyo 152-8550 Japan; 5 Graduate School of Kuroshio Science, Kochi University, 2-5-1 Akebono-cho, Kochi, 780-8520 Japan; 6 Biology Department, University of San Carlos, 6000 Nasipit, Talamban, Cebu City, Philippines; 7 Research Laboratory of Hydrothermal Chemistry, Faculty of Science, Kochi University, 2-17-47 Asakurahonmachi, Kochi, 780-8073 Japan; 8 Education and Research Center for Aquascience, Faculty of Bioindustry, Tokushima University, 96-14 Seto, Dounoura, Naruto, Tokushima 771-0361 Japan

**Keywords:** Biological techniques, Physiology, Plant sciences

## Abstract

Microalgae with high growth rates have been considered as promising organisms to replace fossil resources with contemporary primary production as a renewable source. However, their microscopic size makes it hard to be harvested for industrial applications. In this regard, multicellular macroalgae are more suitable for harvesting. Here, we show that *Ulva meridionalis* has the highest growth rate ever reported for a multicellular autotrophic plant. Contrasted to the known bloom-forming species *U. prolifera* growing at an approximately two-fold growth rate per day in optimum conditions, *U. meridionalis* grows at a daily rate of over fourfold. The high growth ability of this multicellular alga would provide the most effective method for CO_2_ fixation and biomass production.

## Introduction

Microalgae and marine macroalgae (seaweeds) represent the most promising producers of renewable biological resources from carbon dioxide and inorganic nutrients by photosynthesis for the sustainable circular bioeconomy^1,2^. Initially in order to utilize their high growth rates and without using valuable arable land for farming, microalgae were explored to optimize the economics of the application process^3^. However, the harvesting process of microalgae is still a major problem, accounting for about 20–30% of the biomass production cost^4^. The main reasons for the high costs are the small size of microalgae and their culture in dilute media with densities close to that of water, making it difficult to separate the microalgae from the medium. There is currently no microalgal harvesting method that is both efficient and economically viable^4^. By contrast, seaweeds are much simpler to utilize because they can be harvested using a net or similar structure. Furthermore, the green seaweed *Ulva* having a growth rate nearly equal to microalgae can compete with microalgal production^5^. *Ulva* have a large biomass in coastal regions all over the world^6^. Particularly, *U. prolifera* commonly dominates in temperate brackish estuaries, having an ability to tolerate a wide range of salinities^7^. Some variants of this species cause spectacular blooms called green tides, covering several hundreds of kilometers of coastal waters only in a few months^8,9^. The rapid initial expansion of these blooms could be explained by the high growth rates of 10–37% increment per day in the field or under laboratory conditions^10^. From the viewpoint of industrial application, such a high growth rate of *Ulva* is the essential key for algal bioremediation and biomass production for sustainable feed, fuel and chemical generation^5,11,12^.

The Yoshino River estuary on Shikoku Island supports Japan’s largest production of *U. prolifera* by setting out culture nets in winter which yearly attains 60–70 dry-ton as edible green powder^13^. There is also another *Ulva* species occasionally blooming in summer^14^. This species is like *U. prolifera* having a thin and branched morphology. However, based on the microscopic cellular morphology and comparison of a DNA marker, it has been identified as a new species designated as *U. meridionalis* in 2011^15^. Through our field observations of the excessive growth of these two species even in highly variable temperature and salinity conditions in the Yoshino River estuary (Supplementary Fig. 1 and Supplementary Table 1), they would be expected to have high growth potential. Here we examined which conditions are optimal for their growth and report that *U. meridionalis* has an extremely high growth rate.

**Figure. 1:**
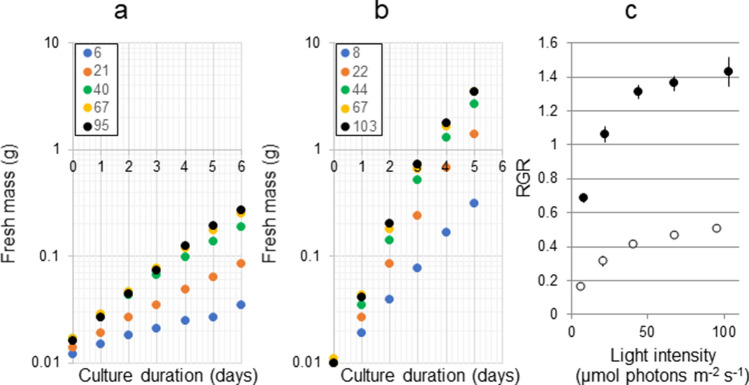
Determination of light intensity saturation for growth in *Ulva prolifera* and *U. meridionalis*. (**a)** *U. prolifera* at 20 °C. (**b**), *U. meridionalis* at 25 °C. Numerical values in key show light intensities (µmol photons m^−2^ s^−1^). (**c**) Changes in RGRs to light intensity gradient. The RGRs (*n* = 3) were calculated from 4 consecutive samples linearly arranged between 0.01 and 1 g in (**a)** and (**b)**. Open circle, *U. prolifera*. Filled circle, *U. meridionalis*. Bar is standard error.

**Table 1:** The RGRs of *Ulva prolifera* and *U. meridionalis* at each temperature and salinity condition for the optimum growth. In addition to the RGR data in Fig. 2, further RGRs were measured of 2 and 5 seedling stocks of different generations and expressed as mean ± s.d. and range.

Species (optimum growth condition)	RGR
*Ulva prolifera* (20 °C, salinity 5)	0.81 ± 0.098, 0.70–0.89 (*n* = 3)
*Ulva meridionalis* (30 °C, salinity 30)	1.41 ± 0.081, 1.28–1.46 (*n* = 6)

## Results

For determination of light intensity saturation for growth in *U. prolifera* and *U. meridionalis*, five light intensities were initially tested. Consecutive data of the fresh mass values from 0.01 to 1 g in our experimental setup (Supplementary Fig. 2) were logarithmically transformed and linearly arranged in each of the light conditions, indicating that the two species grow exponentially (Fig. 1a,b). However, the relative growth clearly declined when the fresh mass was over 1 g due to self-shading (Fig. 1b). Therefore, relative growth rates (RGRs) were calculated from the consecutive fresh mass values of < 1 g and plotted in relation to the light intensities (Fig. 1c). Light intensities of > 67 µmol photons m^−2^ s^−1^ gave saturated growth. Consequently, light intensity of 100–200 µmol photons m^−2^ s^−1^ was used as the standard light condition for all other experiments.

**Figure. 2:**
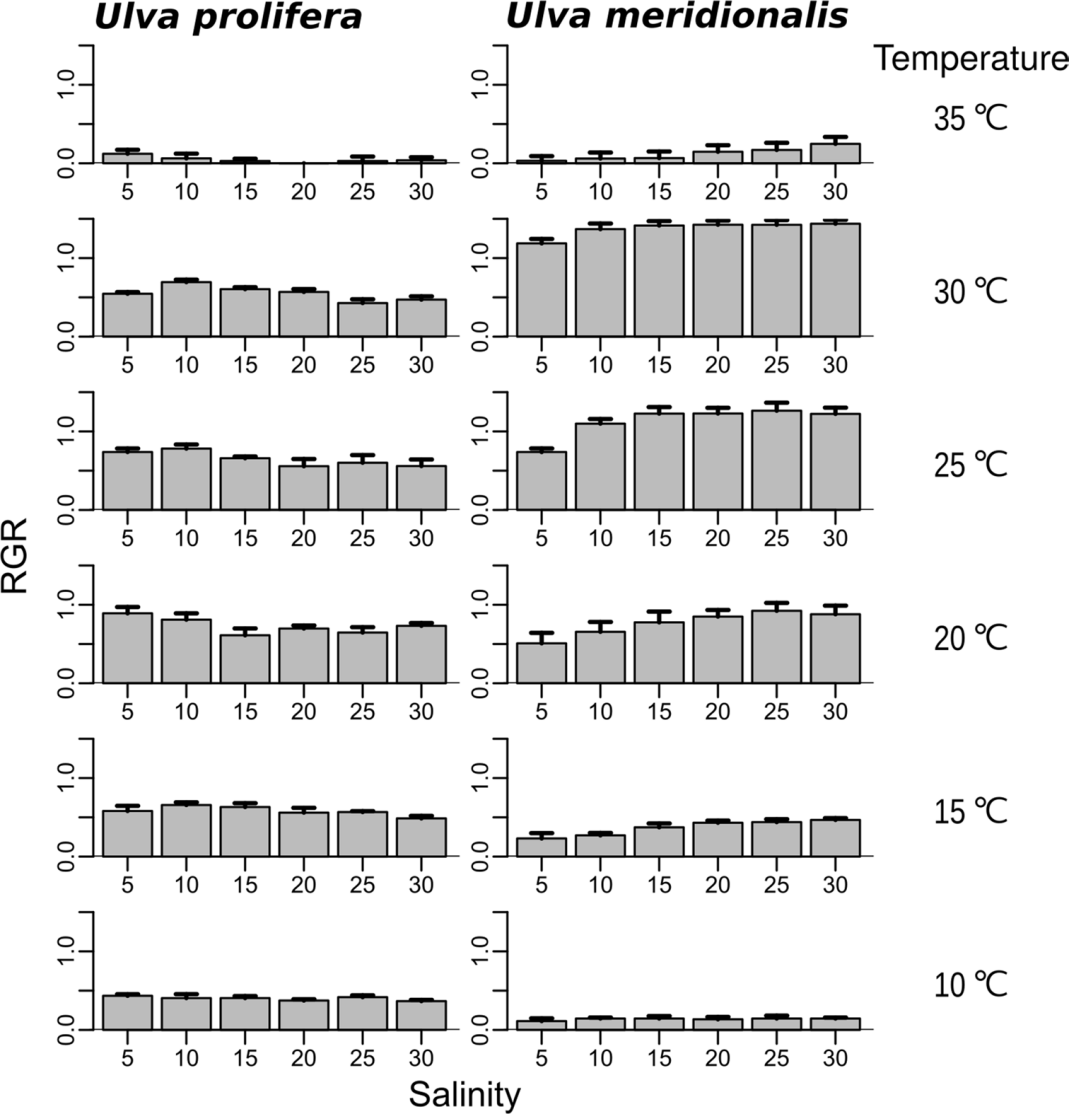
Growth characteristics in various combinations of temperature (10–35 °C) and salinity (5–30) of *Ulva prolifera* and *U. meridionalis*. RGRs in each combination (*n* = 4 in *U. prolifera*, *n* = 3 in *U. meridionalis*). The average RGRs (column) were calculated from consecutive fresh mass samples between 0.01 g and 1 g as in Fig. 1. Bar is standard error.

**Figure. 3:**
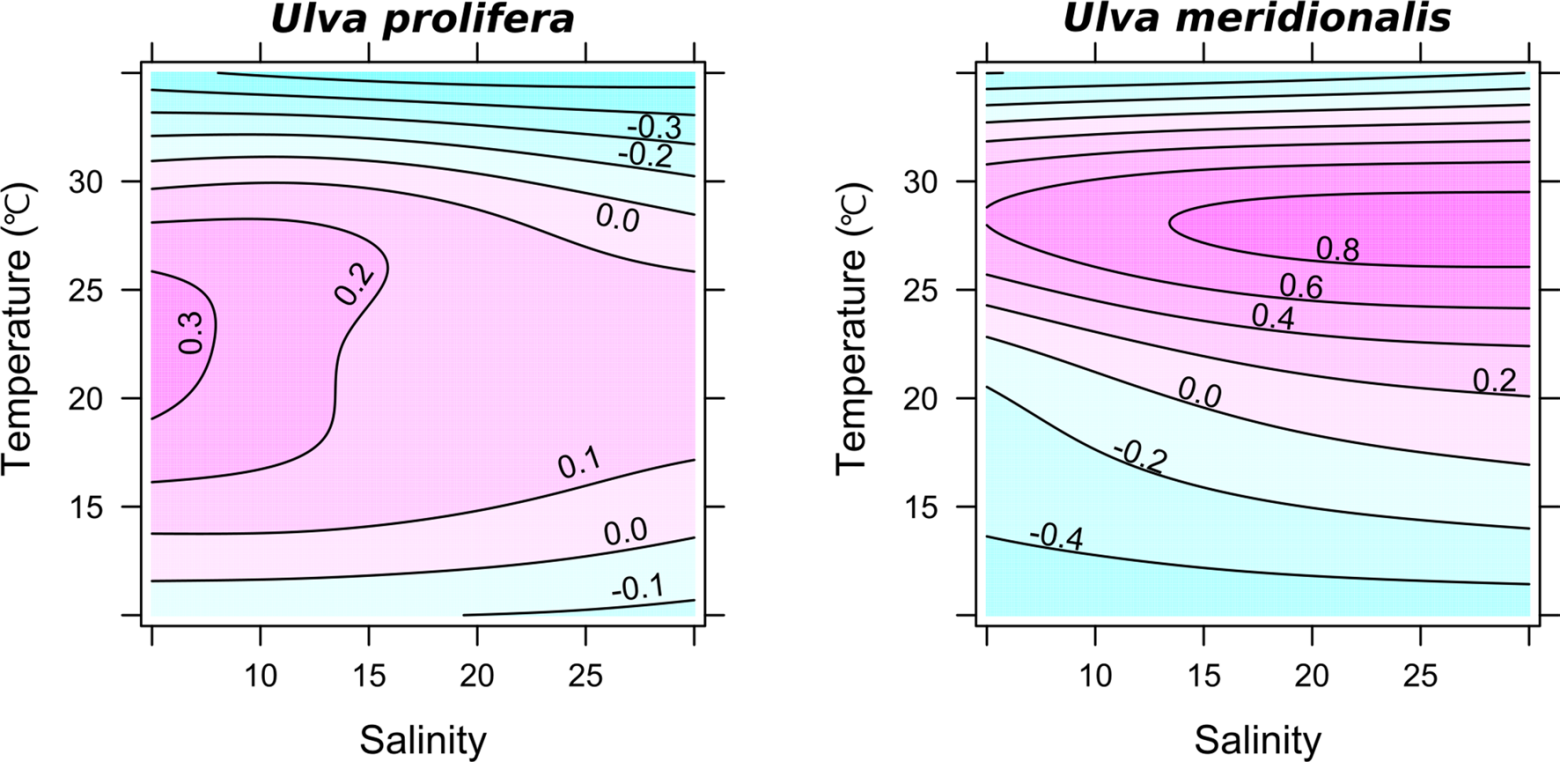
Estimation of optimal growth conditions by the generalized additive model using the data in Fig. 2. The areas (°C × psu) of the optimal growth conditions for temperature and salinity (≥ 0 = average RGR) were detected using the thin plate smoothing splines and 456.23 for *U. prolifera* and 348.80 for *U. meridionalis*.

**Figure 4:**
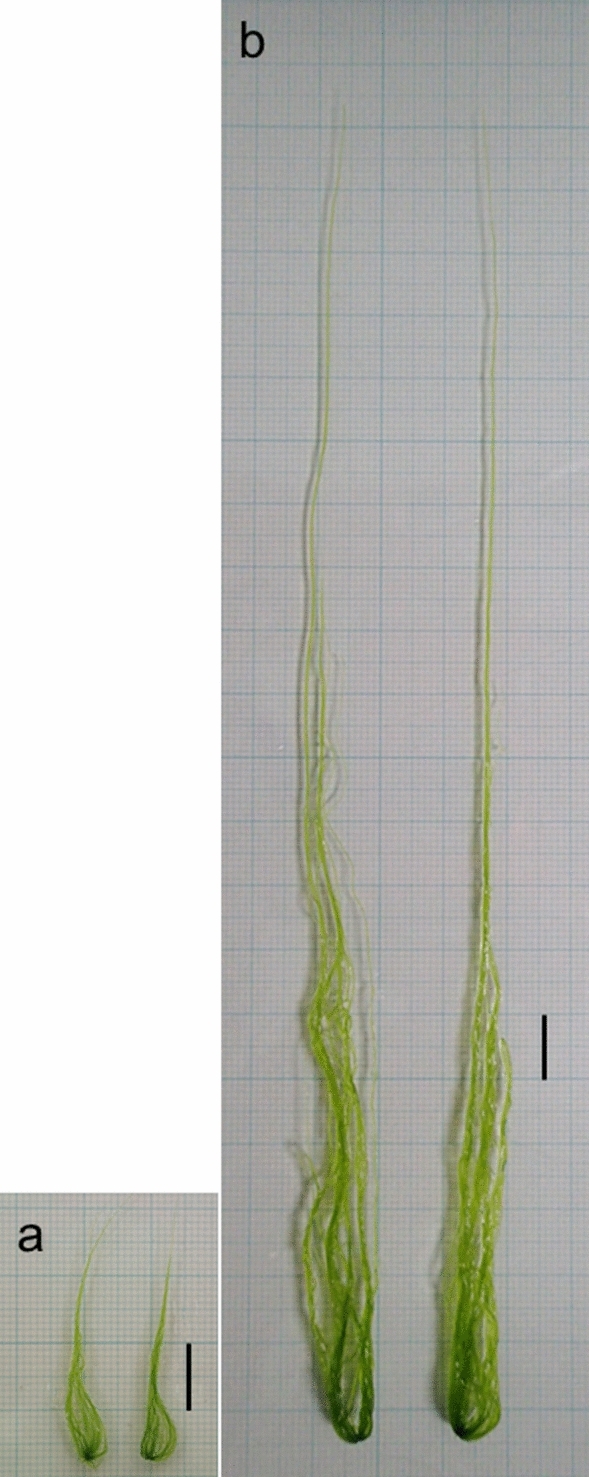
The maximum growth of thallus clusters of *U. meridionalis*. (**a**) The early growth stage. (**b**) The same thallus clusters after 48 h of culture at 30 °C and salinity 30. Scale bars, 1 cm (**a**,**b**).

The RGRs of *U. prolifera* and *U. meridionalis* were obtained at practical ranges of temperature and salinity in their brackish habitats (Figs. 2, 3). *Ulva prolifera* showed consistently high RGRs (0.37–0.89 day^−1^) over broad ranges of salinities 5–30 and temperatures of 10–30 °C. In contrast, *U. meridionalis* showed extremely high RGRs of > 1.4 day^−1^ at salinities 10–30 at 30 °C, although the RGRs clearly declined as temperatures decreased. In the optimum condition of salinity 30 and 30 °C, *U. meridionalis* showed RGR 1.44 day^−1^, increasing 18-fold in fresh mass after 48 h of culture (Fig. 4). To check the intrinsic variability of the highest RGR, additional RGRs were repeatedly measured for samples taken from different generations. The result confirmed that the extremely high RGRs of around 1.4 day^−1^ are stable over generations (Table 1).

Table 2 shows that C content in dry mass was not significantly different between *U. meridionalis* and *U. prolifera*. However, major nutrient contents of N and P in *U. meridionalis* were significantly lower than those in *U. prolifera*.

**Table 2:** Chemical composition of cultured *Ulva prolifera* and *U. meridionalis*. Data are mean (± s.e., *n* = 4). Except for ash and C contents, average values of moisture and N and P contents were significantly different between the two species (*P* < 0.05).

	Moisture (%)	Ash (dry mass %)	C (dry mass %)	N (dry mass %)	P (dry mass %)
*Ulva prolifera*	85.2 ± 0.19	14.75 ± 0.74	35.01 ± 0.37	4.62 ± 0.07	0.59 ± 0.03
*Ulva meridionalis*	81.0 ± 0.58	12.75 ± 0.42	34.86 ± 0.43	3.74 ± 0.08	0.15 ± 0.01

## Discussion

The results imply that *U. prolifera* is a generalist species having a stable growth ability over a wide range of environmental conditions, while *U. meridionalis* is a specialist species showing rapid growth in a narrow range of high temperatures (Fig. 3). The different growth characteristics reasonably correspond with their spatiotemporal growth patterns observed in the habitat. That is, *Ulva prolifera* occurs in a wide range of brackish estuaries with an extensive seasonal period of luxuriant growth, while *U. meridionalis* has extremely rapid growth in a limited area during a short summer period (Supplementary Fig. 1). Because various strains of *U. meridionalis* have been collected in tropical Okinawan islands^15^, the extremely high growth ability would be selected while being distributed in the high temperature environment.

The somatic cells of *Ulva* divide synchronously under standardized conditions once a day^6^. Accordingly, the RGRs of 0.37–0.89 day^−1^ in *U. prolifera* (Fig. 1a) are equal to a 1.4–2.4-fold increase per day, indicating that almost all the cells divide once a day. The highest RGRs ever reported in autotrophic multicellular algae are 1.03 day^−1^ and 1.00 day^−1^ (reported as 179.2 and 172.7% increment per day) in *U. prolifera* and *U. linza*, respectively^16^, around 0.67 day^−1^ in *U. tepida*^17^ and 0.68 day^−1^ in a filamentous *Chaetomorpha* species causing green tides in tropical waters^18^, which are similar RGRs as in *U. prolifera* in the present study. However, the maximum RGR of 1.41 day^−1^ in *U. meridionalis* (Table 1) means a 4.1-fold daily increase, suggesting that all the cells would divide at least twice a day. Two consecutive cell divisions per day have been reported in microscopic *Ulva* germlings for the early developmental stage during a single dark phase under ordinary light: dark cycle conditions^19^. However, *Ulva meridionalis* cells seem to be able to divide twice a day even in well-developed thalli as in Fig. 4.

Carbon content in dry mass was almost the same in *U. meridionalis* and *U. prolifera* (Table 2), demonstrating that *U. meridionalis* quickly builds up its plant body without reducing the carbon mass percentage and essentially has a carbon fixation ability twice as high as *U. prolifera*. Nevertheless, N and P contents in *U. meridionalis* were clearly lower than those in *U. prolifera*, especially the P content being only a quarter. The typical C to N to P stoichiometry by moles for algal biomass is C_106_: N_16_: P_1_, generally referred to as the Redfield ratio^20^. This average stoichiometry allows quantitative predictions to be made about the quantities of C, N and P required for algal production^21^. Our calculated value of C_154_: N_17_: P_1_ in *U. prolifera* is comparatively close to the typical stoichiometry. However, *U. meridionalis* has a much higher ratio of C to nutrients (= C_595_: N_55_: P_1_), implying that a larger biomass production would be effectively gained even with a lower nutrient supply, particularly for P. In our preliminary culture experiment using an outdoor tank with an upper one square meter of open area continuously supplied with natural seawater adjusted to a concentration of 20–30 µM nitrate and 2–3 µM phosphate, this species showed a high productivity of approximately 60 g-dry m^−2^ day^−1^ (ref. 14). Commercially prosperous *Ulva* production rates have been estimated to be 20–26 g-dry m^−2^ day^−1^ over a full year from pond raceway systems in South Africa^5^. If the same systems are operated in tropical regions in which seawater with the optimal high temperature for *U. meridionalis* can be constantly supplied, the production rates would be 2–3 times by using *U. meridionalis*. Biomass productivities of common industrial microalgae such as *Chlorella* and *Spirulina* have been reviewed to be 11–69 g-dry m^−2^ day^−1^ for open pond production systems or closed photobioreactors^22^. We have acquired a unique multicellular algal strain with easy handling for harvest and, in addition, having a high productivity nearly equal to the maximum values of microalgae. *Ulva meridionalis* cultivation would be one of the most effective options for CO_2_ fixation and biomass production in the future.

## Methods

### Strains and preparation of seedling stocks

The *U. prolifera* strain E18^23^ and *U. meridionalis* strain E16^14,15^ respectively maintained as unialgal isolates in Usa Marine Biological Institute, Kochi University were used. Their seedling stocks for the growth experiments were prepared according to the ‘germling cluster’ method for unattached macroalgal culture in the free-floating form^24^. Synchronous zoid formation in each strain was induced by cutting a well-developed thallus into small fragments of 1–2 mm length. Several tens of the fragments were cultured in a Petri dish containing 40 mL of enriched natural seawater (ES) medium^25^ at 20 °C for *U. prolifera* or 25 °C for *U. meridionalis* with a 12 h:12 h L:D cycle at 100–200 µmol photons m^−2^ s^−1^. Under these conditions, thallus fragments released zoids within 3 days. Aliquots of the zoid suspension densely concentrated using their phototactic response were placed in Petri dishes, adjusted to a density of > 10^4^ zoids per 1 mL medium, and incubated under the same condition as mentioned above. After 2–3 weeks, germlings grew at a high density on the bottom of the dish and attached to one another to form aggregations that appear like a green mat. The aggregations were scraped off the dish, torn into numerous small clusters of germlings and cultured with aeration, drifting freely with the current in a vessel. When they attained a length of 1 mm or more, they were statically stocked under weak light (12 h:12 h L:D cycle at < 50 µmol photons m^−2^ s^−1^) at 20 °C until being used for the growth experiments.

### Relative growth rate measurement

To reduce the lag phase caused by the inactive condition of the stocked materials, hundreds of the germling clusters were pre-cultured in a round 1L-flask with continuous aeration for several days. The flask was filled with 1/2 ES medium for which half the amount of the enrichment solution for the standard ES medium was added to artificial seawater adjusted to salinity 15 (Supplementary Table 2). Temperature and light conditions were set as above for the germling growth condition. The medium was exchanged every day. When the thalli of the clusters grew to 5–10 mm in length in this pre-culture, they were subsequently cultured in 500 mL-flasks at various experimental conditions set in the incubator (Supplementary Fig. 2).

Five light intensities from 6 to 103 µmol photons m^−2^ s^−1^ with a 12 h:12 h L:D cycle were provided by placing optical neutral filters (ND filter, Fuji Film, Tokyo, Japan) between the light source and the water bath in the setup (Supplementary Fig. 2). Light intensity was measured at the bottom of the flask with a LI-190SA quantum sensor (Li-Cor Biosciences, Lincoln, NE, USA). Around 0.01 g fresh mass of the thallus clusters was initially set in the flask filled with 500 mL of the 1/2 ES medium (Salinity 32) which was exchanged every other day. In order to determine the fresh mass of the living materials without causing damage by drying, the thallus clusters were held between sterilized paper towels more than five times to carefully remove water on the surface, immediately put in a Petri dish (6 cm in diameter) filled with each medium on the balance, quantified and returned to the same culture condition. This mass measurement was made within a few minutes at the end of the light period every day, equally spaced at 24 h-intervals. Relative growth rate expresses the continuously accelerating growth of algae during the exponential phase, represented by RGR = (ln W_1_—ln W_0_) day^−1^ in which W_0_ is the initial fresh mass in the culture at zero time, W_1_ being the mass after 24 h. The RGRs of *U. prolifera* and *U. meridionalis* were measured in a total of 36 conditions of various salinities (5, 10, 15, 20, 25, 30) and temperatures (10, 15, 20, 25, 30, 35 °C) under the standard light condition (100–200 µmol photons m^−2^ s^−1^). To confirm the stability of the highest growth performance, seedlings from different generations were produced by repeating subculture through zoids and their RGRs were measured at the optimum growth condition for each species.

### Elemental composition of algal biomass

For analyses of elemental composition of the cultured *Ulva*, initial fresh mass of 0.01 g of *U. prolifera* and *U. meridionalis* were cultured in 1L-flasks filled with 1/2 ES medium at 20 °C and 25 °C, respectively. The medium was exchanged every other day. The samples were harvested after 6 days of culture, when they were in exponential growth. The samples were repeatedly rinsed with distilled water to remove residual salts from the culture medium and surface moisture was carefully removed with paper towels. Average fresh mass of the samples harvested from four replicates (*n* = 4) were 0.195 (± 0.009 s.e.) g for *U. prolifera* and 0.320 (± 0.006 s.e.) g for *U. meridionalis*. The water content of the fresh mass was determined by the weight difference before and after freeze drying of the thalli. The carbon (C) and nitrogen (N) content in the dried thalli was measured using CHNS analyzer (Flash EA, Thermo Fischer Scientific Inc. MA, USA). Phosphorus (P) content was determined by ICP-AES (Optima 4,300 DV CYCRON, PerkinElmer Inc., MA, USA).

### Statistical analysis

Optimal growth conditions for *U. prolifera* and *U. meridionalis* were estimated from the RGR data in the tested combinations of temperature and salinity using thin plate smoothing splines of the generalized additive model. Then, we defined conditions indicating average and higher RGRs as the optimal growth conditions. Differences between the two species in chemical components were assessed with unpaired t-test.

## Supplementary information

Supplementary information.
