# Supplementary material for: Fourfold daily growth rate in multicellular marine alga Ulva meridionalis
Source: Sci Rep. 2020 Jul 28;10:12606. doi: 10.1038/s41598-020-69536-4 (PMC7387555; doi:10.1038/s41598-020-69536-4)
Supplement: Supplementary file 1 — Supplementary information. [file 41598_2020_69536_MOESM1_ESM.pdf]

Fourfold daily growth rate in multicellular marine alga *Ulva meridionalis*

Masanori Hiraoka[1,5]*, Yutaro Kinoshita[2], Motoki Higa[3], Shuntaro Tsubaki[4], Alvin P. Monotilla[5,6], Ayumu Onda[7], Akinori Dan[8]

[1]Usa Marine Biological Institute, Kochi University, 194 Inoshiri, Usa, Tosa, Kochi 781-1164, Japan; [2]MSc Science Department, Graduate School of Integrated Arts and Science, Kochi University, 2-5-1 Akebono-cho, Kochi 780-8520, Japan; [3]Laboratory of Plant Ecology, Faculty of Science and Technology, Kochi University, 2-5-1 Akebono-cho, Kochi 780-8520, Japan; [4]Department of Chemical Science and Engineering, School of Materials and Chemical Technology, Tokyo Institute of Technology, 2-12-1 Ookayama, Meguro, Tokyo 152-8550, Japan; [5]Graduate School of Kuroshio Science, Kochi University, 2-5-1 Akebono-cho, Kochi 780-8520, Japan; [6]Biology Department, University of San Carlos, Nasipit, Talamban, Cebu City 6000, Philippines; [7]Research Laboratory of Hydrothermal Chemistry, Faculty of Science, Kochi University, 2-17-47 Asakurahonmachi, Kochi 780-8073, Japan; [8]Education and Research Center for Aquascience, Faculty of Bioindustry, Tokushima University, 96-14 Seto, Dounoura, Naruto, Tokushima 771-0361, Japan

*e-mail: mhiraoka@kochi-u.ac.jp

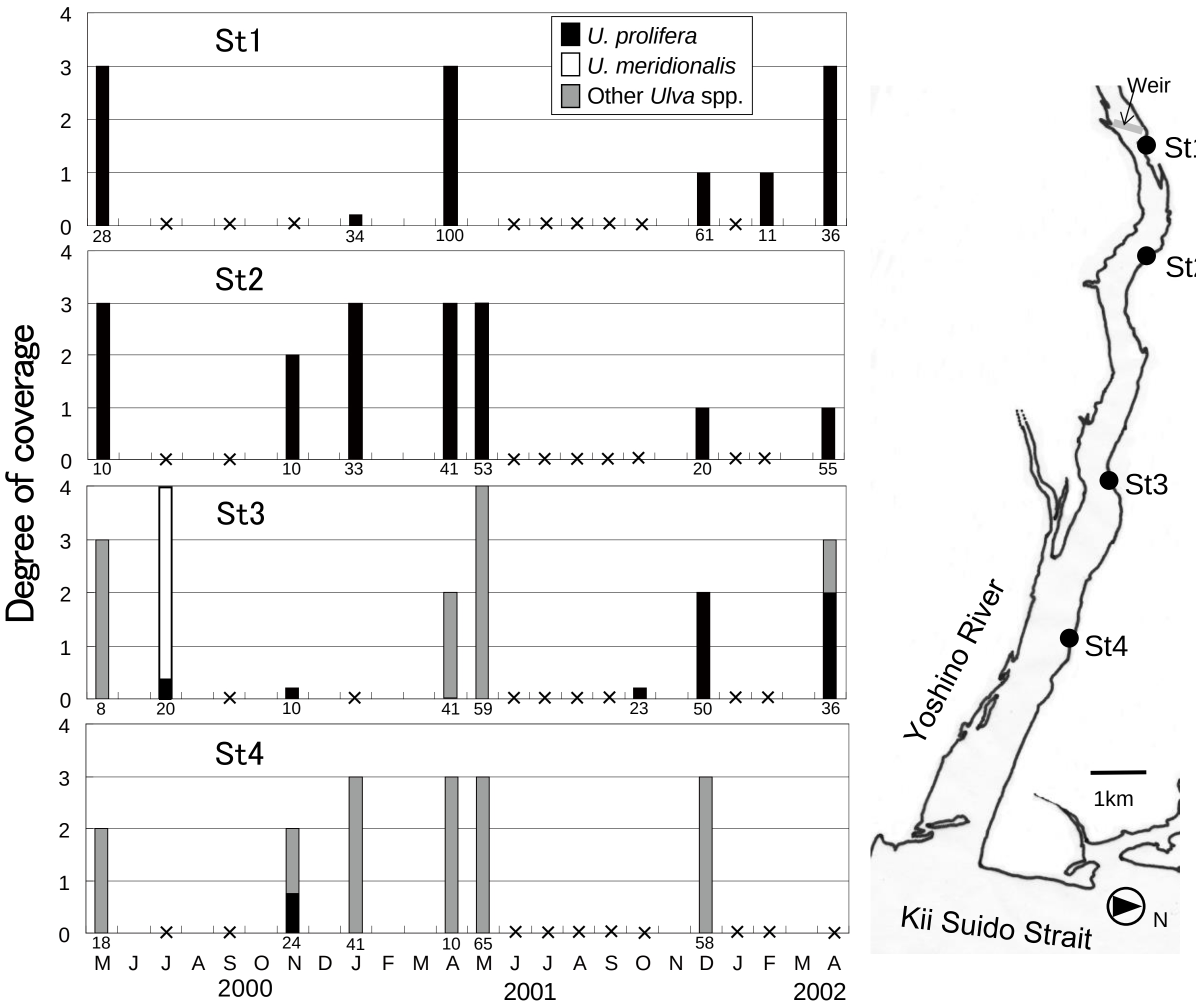

Supplementary Fig. 1. | Spatiotemporal distribution pattern of *Ulva* spp. in the Yoshino River estuary, Shikoku Island, Japan.

The field observations of *Ulva* population were conducted monthly or bimonthly from May 2000 to April 2002 at 4 sites of St1-4 in the Yoshino River estuary. There is an old weir (situated above St1) at 14 km upstream from the river mouth, which allows freshwater to flow down but prevents seawater for going up at the flood and makes polyhaline conditions (Salinity about 20) in the downstream estuary. During the lowest tide in the study month, a quadrat of 0.5 m × 0.5 m was set down on *Ulva* vegetation area at each site. Abundances of the *Ulva* population were visually estimated as coverage and expressed in a semi-quantitative scale (×: no sample, 0.2: 1-5% coverage, 1: 6-25% coverage, 2: 26-50% coverage, 3: 51-75% coverage, 4: 76-100% coverage). To investigate the species composition in the *Ulva* population, well-developed *Ulva* individuals (n = 8-100) were haphazardly sampled at each collection and number of individuals are indicated below bars. Morphology of cultured thalli of which zoids were isolated from each individual was observed by photomicroscope for species identification. Branched samples having mostly a single pyrenoid per cell were identified as *U. prolifera.* Branched samples with one to four pyrenoids were identified as *U. meridionalis*. Other unbranched samples including at least three species of *U. linza*, *U. californica* and *U. aragoënsis* were treated as an *Ulva* species complex. The *U. prolifera* strain E18 having an obligate asexual life history was collected at St2 on 23 May 2001 and its biflagellate asexual zoids were isolated. Strains of *U. meridionalis* collected at St3 on 18 July 2000 were male and female gametophytes, having a sexual life cycle alternating with isomorphic sporophytes for subculture. Zygotes formed by fusion of their male and female gametes were isolated using their phototactic response and named as E16.

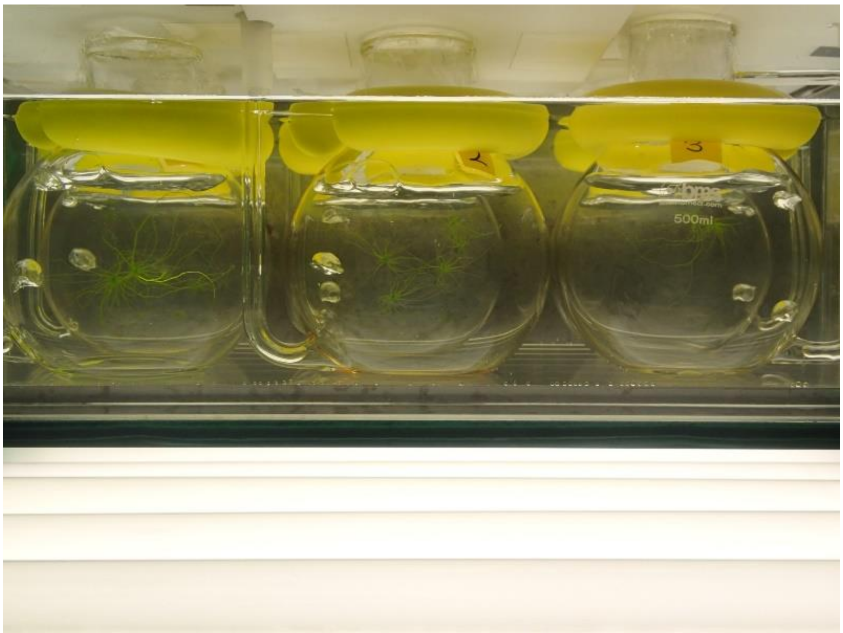

Supplementary Fig. 2. | Setup for measurement of algal RGRs.

The setup was equipped in an incubator (MIR-254, Panasonic, Osaka, Japan). Round glass 500mL-flasks were set in a transparent acrylic box filled with fresh water (= water bath) by which the temperature was kept constant within ±1 ºC and irradiated from the bottom by five white fluorescent tubes (FL20SS·ENW/18HF, Panasonic, Osaka, Japan) arranged in parallel. Initially, around 0.01 g fresh mass of the thallus clusters were put in the 500mL-flask filled with the 1/2 ES medium and continuously agitated with aeration. The medium was exchanged every other day.

Supplementary Table 1. | Temperature and salinity of surface water at each sampling site in the Yoshino River estuary, Shikoku Island, Japan.

Temperature and salinity were measured using a digital thermometer (SK-250WP, Sato, Tokyo, Japan) and a salinometer (T.S Digital Lab., Turumi Seiki, Yokohama, Japan) at St1-4 (see Supplementary Fig. 1) when samplings were conducted.

Date		23 May 2000	18 Jul. 2000	27 Sep. 2000	27 Nov. 2000	25 Jan. 2001	10 Apr. 2001	23 May 2001	26 Jun. 2001	26 Jul. 2001	29 Aug. 2001	21 Sep. 2001	31 Oct. 2001	18 Dec. 2001	29 Jan. 2002	28 Feb. 2002	25 Apr. 2002
Temp. (ºC)	St1	26.1	29.4	21.6	14.4	7.6	18.3	19.9	22.8	28.9	25.8	23.1	18.3	10.3	9.7	11.2	19.2
	St2	24.0	29.3	20.7	14.3	6.6	17.8	20.9	24.7	29.8	26.4	22.6	16.3	9.9	8.1	10.4	18.1
	St3	24.4	28.0	23.3	16.2	6.9	19.3	20.6	26.2	29.9	27.8	23.0	18.6	10.9	8.8	10.4	17.8
	St4	23.4	26.1	24.7	16.9	7.5	16.1	19.4	26.4	28.8	26.8	23.4	17.4	11.5	8.6	9.9	16.5
Salinity	St1	10.4	1.4	0.1	1.3	13.6	12.1	0.3	0.1	0.1	1.1	0.7	0.3	14.2	1.6	11.4	20.0
	St2	23.2	15.5	3.2	3.3	20.7	25.0	17.0	0.5	12.1	5.9	2.5	0.3	22.3	12.0	25.8	21.6
	St3	24.7	22.2	11.1	13.7	27.5	26.7	21.8	1.6	23.5	17.7	4.8	3.8	24.4	19.7	27.7	23.1
	St4	23.0	28.9	20.4	23.4	29.6	28.7	26.1	3.8	26.6	24.4	11.3	5.4	27.8	25.9	29.5	27.5

Supplementary Table 2. | Composition and salinity of artificial seawater.

To make artificial seawater, powder of Marine Art SF-1 (Tomita Pharmaceutical, Naruto, Japan) was dissolved in 25 L distilled water. This chemical composition is disclosed by Tomita Pharmaceutical. Salinity was measured by a YSI-85 salinometer (YSI, Yellow Springs, OH, USA). To provide media with various salinities, this artificial seawater was diluted with distilled water.

Component	Mass L^{-1}
NaCl	22.1 g
$MgCl_2·6H_2O$	9.9 g
$CaCl_2·2H_2O$	1.5 g
Na_2SO_4	3.9 g
KCl	0.61 g
$NaHCO_3$	0.19 g
KBr	96 mg
$Na_2B_4O_7·10H_2O$	78 mg
$SrCl_2$	13 mg
NaF	3 mg
LiCl	1 mg
KI	81 µg
$MnCl_2·4H_2O$	0.6 µg
$CoCl_2·6H_2O$	2 µg
$AlCl_3·6H_2O$	8 µg
$FeCl_3·6H_2O$	5 µg
$Na_2WO_4·2H_2O$	2 µg
$(NH_4)6Mo_7O_{24}·4H_2O$	18 µg
Salinity	32.0
